# What are the predictors of hand hygiene compliance in the intensive care unit? A cross-sectional observational study

**DOI:** 10.1177/17571774211033351

**Published:** 2021-08-28

**Authors:** Caoimhe Madden, Sinéad Lydon, Chloe Walsh, Emily O’Dowd, Susan Fox, Akke Vellinga, Kathryn Lambe, Omar Tujjar, Cathriona Greally, Michael Power, John Bates, Paul O’Connor

**Affiliations:** 1 Department of General Practice, School of Medicine, National University of Ireland, Galway, Ireland; 2 Irish Centre for Applied Patient Safety and Simulation; 3 School of Medicine, National University of Ireland, Galway; 4 Health Services Executive, Dublin, Ireland; 5 Sligo University Hospital, Sligo, Ireland; 6 Galway University Hospital, Galway, Ireland; 7 Critical Care Programme, National Clinical Programmes, Health Services Executive, Dublin, Ireland

**Keywords:** Hand hygiene, critical care, infection control, behaviour, audit

## Abstract

**Background:**

Although appropriate hand hygiene (HH) practices are recognised as the most effective preventative strategy for infection, adherence is suboptimal. Previous studies in intensive care units (ICUs) have found differences in HH compliance between those moments that protect the patient, and those that protect the healthcare provider. However, such studies did not control for other variables known to impact HH compliance.

**Aim:**

To examine HH among healthcare workers (HCWs) in ICU settings, and identify whether there is a statistical difference in HH compliance between patient-protective and self-protective moments, while controlling for other variables known to influence HH compliance (i.e. professional role, unit and shift time).

**Methods:**

A cross-sectional observational study was conducted in four ICUs across three Irish hospitals. Compliance was assessed according to the WHO’s ‘five moments for hand hygiene’. HCW professional role, total number of ‘opportunities’ for HH and whether compliance was achieved were recorded.

**Results:**

A total of 712 HH opportunities were recorded, with an overall compliance rate of 56.9%. Logistic regression analysis revealed that physicians, allied healthcare professionals and auxiliary staff were less likely than nurses to engage in HH. HCWs were more likely to comply during night shifts compared to morning shifts, and with self-protective as compared to patient-protective HH moments.

**Conclusion:**

The information provided in this study provides a data-driven approach that ICUs can use to tailor HH interventions to where, when and for whom they are most required.

## Background

Healthcare-associated infections (HCAIs) are associated with increased morbidity, prolonged hospital stays and a high number of in-hospital deaths (Herwaldt et al., 2006). Appropriate hand hygiene (HH) practices are the most effective preventative strategy for HCAIs (Pittet et al., 2006), and are particularly important in intensive care units (ICUs) where patients are critically ill, immuno-compromised and particularly vulnerable to HCAIs (Hughes, 2008). Accordingly, the World Health Organization (WHO) has developed HH guidelines that outline the ‘five moments’ when HH is essential for protecting the healthcare worker (HCW) and the patient (WHO, 2009). These guidelines have been widely adopted in healthcare settings and are considered highly influential in improving HH practices. However, adherence to HH practices has continued to be suboptimal. A recent systematic review (Lambe et al., 2019) examining HH compliance across 61 ICU-based studies reported average compliance of 59.6% internationally.

Such data highlight a pressing need to develop an improved understanding of factors influencing HH in order to inform the development of appropriate and effective improvement strategies. Previous research has suggested that variables such as professional role and shift pattern impact HH compliance. Physicians have been found to comply less frequently with HH guidance than their allied health and nursing counterparts (Süzük et al., 2015). Further, significantly lower rates of HH compliance have been observed during morning shifts compared with evening and night shifts (AlNakhli et al., 2014; Kouni et al., 2014; Rosenthal et al., 2013).

It has also been suggested that levels of compliance differ across the WHO five moments of HH. HCWs are more likely to engage in HH after exposure to body fluids, and after contact with the patient and their surroundings, than before patient contact or before an aseptic task (Stahmeyer et al, 2017; Süzük et al., 2015). However, these assertions are based on limited empirical evidence. Among the relatively small number of studies that provide such data, reporting is limited to descriptive statistics (e.g. Chavali et al., 2014) or univariate statistical analyses (e.g. Stahmeyer et al., 2017; Süzük et al., 2015). These analyses do not control for those variables outlined in the previous paragraph that are known to influence HH compliance. Therefore, the aim of the current study was to examine HH compliance rates in ICU settings, and identify whether there is a statistical difference in compliance between those HH moments that primarily protect the patient (i.e. Moment 1 and 2), and those that primarily protect the healthcare provider (i.e. Moments 3, 4 and 5). The rationale for grouping the moments as patient protective versus provider protective is because HH before patient contact (Moment 1) and before an aseptic procedure (Moment 2) are the moments that are most closely related with HCAI transmission in patients, compared to the other three moments (Süzük et al., 2015). Further, unlike in previous studies, logistic regression analysis will be used to control for variables known to influence HH compliance (i.e. healthcare profession, setting, shift time). The implications of these findings for the design of HH intervention and auditing are discussed.

## Methods

### Design

A cross-sectional observational study was conducted across four hospital units between February 2017 and June 2018.

### Setting

The study was carried out in four units (three general ICUs, one high-dependency unit [HDU]) across three teaching hospitals in the Republic of Ireland. Hospital A had an 11-bed ICU, of which two beds were in isolation rooms, and a six-bed HDU, of which two were in isolation rooms. Hospital B had a five-bed ICU, and Hospital C had a six-bed ICU and four isolation rooms. Staffing ratios were one nurse to one patient in the ICU, and one nurse to two patients in the HDU.

### Ethical approval

Ethical approval was received from the Research Ethics Board of each of the three hospitals in which the observations were completed. As it was considered potentially disruptive to patient care to obtain written informed consent, verbal consent was received from each HCW before observation.

### Participants

All HCWs who came into contact with patients or the patient zone were observed. These included nursing staff (e.g. staff nurses, clinical nurse managers), physicians (e.g. doctors, consultants), allied health professionals (e.g. physiotherapists, radiographers, healthcare assistants) and auxiliary staff (e.g. housekeeping staff, catering staff, porters and IT technicians).

### Behavioural observation

Observers were five health service researchers. Before the beginning of the study, observers were trained in HH observation methods by an infection control practitioner in a university teaching hospital. Training consisted of a presentation covering HCAI transmission awareness, HH moment recognition and recording methods. Further, in order to ensure accuracy of behavioural observation, and good interrater reliability (IRR), ‘practice sessions’ were conducted in a clinical unit, whereby two observers conducted observations together and compared results, with a target IRR of at least 80% for validation of training and results (Artman et al., 2012). These IRR observations were not included in the analysis.

The standardised WHO protocol ‘Five moments for hand hygiene’(WHO, 2009), considered the ‘gold standard’ of HH observation, was applied to assess compliance. The five moments included: (1) before patient contact; (2) before an aseptic task; (3) after body fluid exposure risk; (4) after patient contact; and (5) after contact with patient surroundings.

The WHO HH audit form was modified for the Irish context. Data collected included HCW professional role (i.e. nursing staff, physicians, allied healthcare professionals and auxiliary staff), shift time (i.e. morning, noon, night), setting (i.e. Hosptial A/B/C), opportunity to perform HH and whether compliance was achieved. An ‘opportunity’ was defined as the occurrence of any of the five indications during the observed care sequences. The primary dependent variable was HH compliance, which was achieved by performing either hand washing (with soap and water) or hand sanitizing (with alcohol-based hand rub). Failure to perform HH when indicated resulted in the recording of non-compliance. Overall compliance was calculated as the number of compliant opportunities divided by the total number of opportunities.

The quality of HH performance was not evaluated. When two HH opportunities occurred together, the procedure with the theoretically greater impact for HCAI risk was recorded (e.g. Moment 2 over Moment 1). If there was uncertainty surrounding compliance due to the HCW being partially or fully outside the view of the observer, the opportunity was not recorded.

IRR was established by having observers conduct a portion of the observations together and assessing concordance. IRR was calculated by dividing the number of times independent observers agreed on the outcome of an observation by the total number of opportunities for agreement.

### Procedure

Six 2-h periods of observation were carried out over the course of five days at each hospital, across all three shifts (morning/daytime/night), for a total of 12 h of observation at each of the three participating hospitals (36 h of total observations). HCWs in each ICU were notified in advance of the 2-h periods of observation. Observers maintained a discreet presence, and randomly rotated across bedspaces, spending 15 min at each. Patient bedspaces were not observed if the HCW did not consent to observation, bed curtains were drawn before, or during, the observations, if the patient was receiving palliative care or in the case of an emergency (e.g. cardiac arrest).

### Statistical analysis

Data were analysed using SPSS version 25 (IBM Corp., Armonk, NY, USA). Logistic regression analysis was performed to assess the relationship between a number of factors (specifically professional role, hospital, shift time and HH moment) on HH compliance. The five HH moments were coded as either ‘patient-protective’ (i.e. Moments 1 and 2) or ‘self-protective’ (i.e. Moments 3, 4 and 5). The outcome variable was binary, and coded as ‘1’ if the HCW complied with HH, and ‘0’ if not. The model included variables for each professional role, hospital, time and Moment contrasted against the ‘reference’ (i.e. nursing staff, Hospital A, morning and patient-protective moments).

## Results

A total of 712 HH opportunities were recorded by observers across the four units. The total overall HH compliance was 56.9%. See Table 1 for HH compliance across professional groups, shifts, setting and moments. As can be seen in Table 1, nursing staff had the highest percentage compliance (61.1%) compared to other professional groups. At 70.5%, Hospital A had notably higher compliance than other hospitals (55.3% and 41%, respectively). Compared to other shift patterns, compliance was highest during the night shift (82.6%), and self-protective moments were complied with more frequently (61.7%) than patient-protective moments (42.5%).

**Table 1.** Compliance observed across professional category, setting, shift time and HH moment.

Category	Total opportunities	Compliance	95% CI for compliance
Professional role			
Nursing staff	560	342 (61.1)	57–65
Physician	72	35 (48.6)	37–59
Allied health + auxiliary staff	80	28 (35)	25–46
Setting			
Hospital A	315	222 (70.5)	65–75
Hospital B	141	78 (55.3)	47–63
Hospital C	256	105 (41.0)	35–47
Shift time			
Morning	430	225 (52.3)	48–57
Afternoon	213	123 (57.7)	51–64
Night	69	57 (82.6)	72–90
HH moment			
Patient-protective	179	76 (42.5)	36–50
Self-protective	533	329 (61.7)	58–66
Total	712	405 (56.9)	53–60

Values are given as n or n (%) unless otherwise indicated.

HH, hand hygiene.

### Inter-observer agreement

Over half of the total number of observations (54.5%) were observed concurrently by two observers. Of these observations, agreement was achieved for 92.9% of observations, which was considered adequate inter-observer agreement (Artman et al., 2012).

### Predictors of compliance

Logistic regression analysis revealed that the full model containing all predictors was statistically significant (X^2^ (7, n = 712) = 112.85; *P* < 0.001), indicating that the model was able to distinguish between respondents who complied and did not comply with HH. The model as a whole explained between 14.7% (Cox and Snell R square) and 19.7% (Nagelkerke R squared) of the variance in HH compliance, and correctly classified 65.9% of cases.

As shown in Table 2, the majority of the independent variables made a unique statistically significant contribution to the model. With regards to professional role, it was found that physicians as well as allied health professionals and auxiliary staff were less likely than nurses to engage in HH compliance. HCWs were less likely to comply in Hospital B and C, compared to Hospital A. For shift time, the likelihood of engaging in HH increased later in the day, with HCWs 2.4 times more likely comply during night-time shifts compared to morning shifts. Finally, participants were 2.6 times more likely to comply with HH if the opportunity was self-protective as opposed to patient-protective, controlling for all other factors in the model.

**Table 2.** Logistic regression model for predictors of HH compliance in ICU settings.

Predictor		B	SE	Wald	*P*	OR	95% CI for OR
Professional role	Nurse	Reference					
	Physician	−0.76	0.27	7.74	0.01*	0.47	0.28–0.80
	Allied/auxiliary	−1.40	0.27	26.12	0.000^†^	0.25	0.14–0.42
ICU setting	Hospital A	Reference					
	Hospital B	−0.67	0.23	8.87	0.003*	0.51	0.33–0.80
	Hospital C	−0.1.28	0.20	41.84	0.000^†^	0.28	0.19–0.41
Type of shift	Morning	Reference					
	Day	−0.00	0.18	0.00	0.99	0.99	0.70–1.43
	Night	0.88	0.36	5.88	0.02*	2.41	1.18–4.89
HH moment	Patient-protective	Reference					
	Self-protective	0.95	0.19	24.29	0.000^†^	2.59	1.77–3.78

* *P* < 0.05.

† *P* < 0.001.

HH, hand hygiene; ICU, intensive care unit; OR, odds ratio.

## Discussion

In spite of the recognition of HH as the most important infection control practice, compliance continues to be suboptimal, as reflected in the overall compliance of the current study. In addition to determining HH compliance rates in Irish ICU settings, the aim of this study was to consider variables that may impact compliance. It was found that professional role, setting, shift pattern and type of HH moment all impacted the likelihood of compliance. HH interventions have been suboptimal to date, with poor methodological rigour and much variability in effectiveness (Lydon et al., 2017). Therefore, there is a need to consider those factors that influence HH compliance and use this information to inform targeted intervention and best practice in audit.

Based on the observations carried out in this study, the average overall compliance was 56.9%. This level of compliance is directly comparable to findings from a recent systematic review which derived an estimate HH compliance rate of 59.6% across 61 ICU-based studies (Lambe et al., 2019). This is a notable discrepancy between the overall compliance rate and minimum targets for acceptable HH compliance. Although there is no universally agreed minimum acceptable level of compliance, a number of countries (e.g. Australia, Canada, New Zealand, Ireland) use 80% or 90% as a baseline compliance target, and the WHO recommends that HH role models have compliance of at least 80% (WHO, 2010). It is suggested that there is a need to examine the rationale behind these HH compliance targets.

The highest compliance levels were found among nursing staff, and physicians were the least likely professional group to comply compared to nurses. This is unsurprising; historically, compliance has been much higher in nurses than in physicians (Erasmus et al., 2010; Kouni et al., 2014; Mazi et al., 2013; Scheithauer et al., 2011) and inappropriate attitudes towards HH have been identified among physicians (Erasmus et al., 2009), whereby they perceive a lack of evidence of HH effectiveness in the prevention of HCAIs. There may be a range of underlying factors that influence such attitudes. For example, there is potential that educational activities are primarily targeted towards nurses, while physicians and other HCWs are rarely captured or missed. It has also been suggested that compared to nursing, university programmes for physicians do not include specific training in infection prevention (Musu et al., 2017), resulting in a lack of basic HH training and preventive measures. Further, physicians have reported that they feel strongly influenced to abstain from compliance by negative role models (Erasmus et al., 2009), and frequently adjust their behaviour to match those that they witness in practice. Therefore, a multidisciplinary approach needs to be adapted in preventing transmission of HCAIs (AlNakhli et al., 2014; Tajeddin et al., 2016), whereby innovative educational and behavioural modification strategies place a greater emphasis on professional role, and can be tailored to specific groups of HCWs (Reich et al., 2015; Salemi et al., 2002). Further, educational efforts need to place a greater focus on presenting evidence for HH effectiveness, and incorporating HH into the undergraduate curricula of HCWs. There is also a requirement for positive motivation by proper behaviour of role models of physicians (Snow et al., 2006), which could be achieved by encouraging senior staff members, particularly consultants, to function as role models for junior staff members (Szabó et al., 2015).

HCWs were statistically more likely to comply with HH requirements during the night shift than the morning shift. Similarly, previous research has found that morning or afternoon shifts were associated with significantly lower HH compliance than the night shift (e.g. Kouni et al., 2014; Rosenthal et al., 2013). This difference could be explained by increased ‘traffic’ and crowding in ICUs during morning shifts than night shifts; indeed, there is a confirmed a link between high workload and high demand for HH, and reduced compliance (Pittet, 2000). This highlights the need for audits to be conducted across a range of shifts, and for interventions to be focused on particular shifts to rectify the discrepancies that are associated with time of day.

When controlling for the above factors, it was found that HCWs were more likely to engage in HH practices that protect themselves (e.g. after exposure to body fluid, after patient contact) than those that protect the patient (e.g. before an aseptic task). This finding statistically confirms the tendency of HCWs towards prioritising the protection of oneself from infection rather than patient safety. It has been suggested that in order to improve compliance, attempts should be made to refocus from a self-protection practice to a practice that benefit of self and others (Whitby et al., 2007). A number of HH compliance interventions have been found to be effective in increasing HH compliance in the ICU setting (Lydon et al., 2017). It is suggested that a consideration of practices that benefit HCWs and patients could be emphasised in HH compliance interventions. One potential approach to foster such an approach could be to augment routine HH audits with individual and/or unit-level feedback data to HCWs, supervisors and infection control committees (e.g. Kirkland et al., 2012; Sakihama et al., 2016). Refocusing could also be used to inform education efforts specifically targeted at less compliant groups such as communicating values of others, focus on training other moments or auditing weaker moments more frequently.

### Limitations and strengths

There are a number of strengths associated with this study. First, direct observation was used, which is considered the ‘gold standard’ method of measuring HH compliance (Stewardson and Pittet, 2011). Further, the audit was comprehensive as it observed various HCWs, across multiple shifts, unlike much of the extant research (Lambe et al., 2019). Second, a rigorously standardised HH observation tool with clearly defined standard operating procedures was utilised. Finally, IRR was calculated between multiple observers across a high proportion of opportunities and yielded a high agreement rate in line with recommendations (Artman et al., 2012).

There are also some limitations that should be noted. First, HCWs were aware that the observations were being carried out. This may have resulted in HCWs behaving differently (i.e. complying more) to what they usually would due to being under observation (i.e. the Hawthorne effect (Buchanan and Huczynski, 2013), and has been considered in other HH observational studies (e.g., Kouni et al, 2014). However, a systematic review of over 60 ICU HH compliance studies found similar levels of HH compliance regardless of whether the observation was covert or not (Lambe et al., 2019). Second, the current study did not give consideration to other situational factors or conditions of the working environment that may have impacted on HH compliance, such as patient dependency and acuity, staffing and other features of context. Such factors affect HCW workload, and in turn affect compliance (Pittet, 2000). Finally, some of the samples within categories are relatively small, which will have influenced the generalisability of the findings and resulted in wide confidence intervals. Further, considering the study was carried out solely in an Irish context, the generalisability of findings to other countries may also be questioned. However, the fact that overall compliance is similar to studies conducted across a range of geographical locations (Lambe et al, 2019) provides some support to the generalisability of the findings.

## Conclusion

This study has shown that there are statistically significant differences in HH compliance levels between different professional groups, settings, shifts and moments of HH. Considering the effectiveness of HH interventions has been found to be lower than desirable, it is suggested that a more targeted approach to HH compliance is likely to be more effective than generic unit-wide interventions. The information provided in this study provides a data-driven approach that ICUs can use to tailor HH interventions to where, when and for whom they are most required.

## References

[bibr1-17571774211033351] AlNakhli DJ Baig K Goh A Sandokji H Din SS . (2014) Determinants of hand hygiene non-compliance in a cardiac center in Saudi Arabia. Saudi Medical Journal 35(2): 147–152. 24562513

[bibr2-17571774211033351] Artman K Wolery M Yoder P . (2012) Embracing our visual inspection and analysis tradition: Graphing interobserver agreement data. Remedial and Special Education 33(2): 71–77.

[bibr3-17571774211033351] Buchanan DA Huczynski AA . (2013) Organizational behaviour. London: Pearson.

[bibr4-17571774211033351] Chavali S Menon V Shukla U . (2014) Hand hygiene compliance among healthcare workers in an accredited tertiary care hospital. Indian Journal of Critical Care Medicine 18(10): 689. 25316980 10.4103/0972-5229.142179 PMC4195200

[bibr5-17571774211033351] Erasmus V Brouwer W Van Beeck E Oenema A Daha T Richardus JH Vos MC Brug J . (2009) A qualitative exploration of reasons for poor hand hygiene among hospital workers lack of positive role models and of convincing evidence that hand hygiene prevents cross-infection. Infection Control and Hospital Epidemiology 30(5): 415–419. 19344264 10.1086/596773

[bibr6-17571774211033351] Erasmus V Daha TJ Brug H Richardus JH Behrendt MD Vos MC van Beeck EF . (2010) Systematic review of studies on compliance with hand hygiene guidelines in hospital care. Infection Control Hospital Epidemiology 31(3): 283–294. 20088678 10.1086/650451

[bibr7-17571774211033351] Herwaldt LA Cullen JJ Scholz D French P Zimmerman MB Pfaller MA Wenzel RP Perl TM . (2006) A prospective study of outcomes, healthcare resource utilization, and costs associated with postoperative nosocomial infections. Infection Control and Hospital Epidemiology 28(12): 1291–1298. 10.1086/509827 17152025

[bibr8-17571774211033351] Hughes R . (2008) Patient safety and quality: an evidence-based handbook for nurses. Rockville, MD: Agency for Healthcare Research and Quality. 21328752

[bibr9-17571774211033351] Kirkland KB Homa KA Lasky RA Ptak JA Taylor EA Splaine ME . (2012) Impact of a hospital-wide hand hygiene initiative on healthcare-associated infections: results of an interrupted time series. BMJ Quality & Safety 21(12): 1019–1026. 10.1136/bmjqs-2012-000800 22822243

[bibr10-17571774211033351] Kouni S Kourlaba G Mougkou K Maroudi S Chavela B Nteli C Lourida A Spyridis N Zaoutis T Coffin S . (2014) Assessment of hand hygiene resources and practices at the 2 children’s hospitals in Greece. Pediatric Infectious Disease Journal 33(10): e247–e251. 10.1097/INF.0000000000000376 25361195

[bibr11-17571774211033351] Lambe K Lydon S Madden C Vellinga A Hehir A Walsh M O’Connor P . (2019) Hand hygiene compliance in the ICU: a systematic review. Critical Care Medicine 47(9): 1251–1257. 31219838 10.1097/CCM.0000000000003868

[bibr12-17571774211033351] Lydon S Power M McSharry J Byrne M Madden C Squires JE O’Connor P . (2017) Interventions to improve hand hygiene compliance in the ICU: a systematic review. Critical Care Medicine 45(11): e1165–e1172. 28857850 10.1097/CCM.0000000000002691

[bibr13-17571774211033351] Mazi W Senok AC Al-Kahldy S Abdullah D . (2013) Implementation of the world health organization hand hygiene improvement strategy in critical care units. Antimicrobial Resistance and Infection Control 2(1): 15. 23673017 10.1186/2047-2994-2-15 PMC3673893

[bibr14-17571774211033351] Musu M Lai A Mereu NM Galletta M Campagna M Tidore M Piazza MF Spada L Massidda MV Colombo S Mura P Coppola RC . (2017) Assessing hand hygiene compliance among healthcare workers in six Intensive Care Units. Journal of Preventive Medical Hygiene 58(3): e231. PMC5668933 29123370

[bibr15-17571774211033351] Pittet D Allegranzi B Sax H Dharan S Pessoa-Silva CL Donaldson L Boyce JM . (2006) Evidence-based model for hand transmission during patient care and the role of improved practices. Lancet Infectious Diseases 6(10): 641–652. 17008173 10.1016/S1473-3099(06)70600-4

[bibr16-17571774211033351] Pittet D . (2000) Improving compliance with hand hygiene in hospitals. Infection Control and Hospital Epidemiology 21(6): 381–386. 10879568 10.1086/501777

[bibr17-17571774211033351] Reich JA Goodstein ME Callahan SE Callahan KM Crossley LW Doron SI Snydman DR Nasraway SA Jr. (2015) Physician report cards and rankings yield long-lasting hand hygiene compliance exceeding 90%. Critical Care 19(1): 292. 26271619 10.1186/s13054-015-1008-4 PMC4536705

[bibr18-17571774211033351] Rosenthal VD Pawar M Leblebicioglu H Navoa-Ng JA Villamil-Gómez W Armas-Ruiz A Cuéllar LE Medeiros EA Mitrev Z Gikas A Yang Y . (2013) Impact of the International Nosocomial Infection Control Consortium (INICC) multidimensional hand hygiene approach over 13 years in 51 cities of 19 limited-resource countries from Latin America, Asia, the Middle East, and Europe. Infection Control and Hospital Epidemiology 34(4): 415-423. 23466916 10.1086/669860

[bibr19-17571774211033351] Sakihama T Honda H Saint S Fowler KE Shimizu T Kamiya T Sato Y Arakawa S Lee JJ Iwata K Mihashi M . (2016) Hand hygiene adherence among health care workers at Japanese hospitals: a multicenter observational study in Japan. Journal of Patient Safety 12(1): 11–17. 24717527 10.1097/PTS.0000000000000108

[bibr20-17571774211033351] Salemi C Canola MT Eck EK . (2002) Hand washing and physicians: how to get them together. Infection Control and Hospital Epidemiology 23(1): 32–35. 11868890 10.1086/501965

[bibr21-17571774211033351] Scheithauer S Oude-Aost J Heimann K Haefner H Schwanz T Waitschies B Kampf G Orlikowsky T Lemmen SW . (2011) Hand hygiene in pediatric and neonatal intensive care unit patients: daily opportunities and indication-and profession-specific analyses of compliance. American Journal of Infection Control 39(9): 732–737. 21704425 10.1016/j.ajic.2010.12.020

[bibr22-17571774211033351] Snow M White GL Jr Alder SC Stanford JB . (2006) Mentor’s hand hygiene practices influence student’s hand hygiene rates. American Journal of Infection Control 34(1): 18–24. 16443088 10.1016/j.ajic.2005.05.009

[bibr23-17571774211033351] Stahmeyer J Lutze B Von Lengerke T Chaberny I Krauth C . (2017) Hand hygiene in intensive care units: a matter of time? Journal of Hospital Infection 95(4): 338–343. 10.1016/j.jhin.2017.01.011 28246001

[bibr24-17571774211033351] Stewardson A Pittet D . (2011) Quicker, easier, and cheaper? The promise of automated hand hygiene monitoring. Infection Control and Hospital Epidemiology 32: 1029–1031. 21931254 10.1086/662023

[bibr25-17571774211033351] Süzük S Edis Ç Çalýk A Akdoðan S Ünal S . (2015) The compliance rates of hand hygiene in intensive care unit and surgical services at a state hospital in Turkey. Journal of the Turkish Society of Intensive Care 13(3): 107.

[bibr26-17571774211033351] Szabó R Morvai J Bellissimo-Rodrigues F Pittet D . (2015) Use of hand hygiene agents as a surrogate marker of compliance in Hungarian long-term care facilities: first nationwide survey. Antimicrobial Resistance and Infection Control 4(1): 32. 26236475 10.1186/s13756-015-0069-0 PMC4521470

[bibr27-17571774211033351] Tajeddin E Rashidan M Razaghi M Javadi SS Sherafat SJ Alebouyeh M Sarbazi MR Mansouri N Zali MR . (2016) The role of the intensive care unit environment and health-care workers in the transmission of bacteria associated with hospital acquired infections. Journal of Infection and Public Health 9(1): 13–23. 26117707 10.1016/j.jiph.2015.05.010

[bibr28-17571774211033351] Whitby M Pessoa-Silva C McLaws M-L Allegranzi B Sax H Larson E Seto WH Donaldson L Pittet D . (2007) Behavioural considerations for hand hygiene practices: the basic building blocks. Journal of Hospital Infection 65(1): 1–8. 10.1016/j.jhin.2006.09.026 17145101

[bibr29-17571774211033351] World Health Organization. (2009) WHO guidelines on hand hygiene in health care. Available at: http://whqlibdoc.who.int/publications/2009/9789241597906_eng.pdf (accessed January 2020).

[bibr30-17571774211033351] World Health Organization. (2010) Hand Hygiene Self-Assessment Framework. Available at: https://www.who.int/gpsc/country_work/hhsa_framework_October_2010.pdf?ua=1 (accessed January 2020).

